# Safety and Feasibility of the BYCROSS^®^ Atherectomy Device for the Treatment of Femoropopliteal Arterial Obstructions: Single-Center Short-Term Outcomes

**DOI:** 10.3390/jcm13061809

**Published:** 2024-03-21

**Authors:** Goudje L. van Leeuwen, Reinoud P. H. Bokkers, Job Oldenziel, Richte C. L. Schuurmann, Cornelis G. Vos, Jean-Paul P. M. de Vries

**Affiliations:** 1 Department of Surgery, Division of Vascular Surgery, University Medical Center Groningen, University of Groningen, 9700 RB Groningen, The Netherlands; g.l.van.leeuwen@umcg.nl (G.L.v.L.); r.c.l.schuurmann@umcg.nl (R.C.L.S.); 2 Department of Radiology, University Medical Center Groningen, University of Groningen, 9700 RB Groningen, The Netherlands; 3 Department of Surgery, Martini Hospital, 9728 NT Groningen, The Netherlands

**Keywords:** peripheral arterial disease, femoropopliteal, superficial femoral artery, popliteal artery, endovascular therapy, atherectomy

## Abstract

**Background:** Endovascular techniques have gained preference over peripheral arterial bypass surgery due to their minimally invasive nature; however, endovascular treatments often show limited efficacy in arterial segments with a high atherosclerotic load. The use of atherectomy devices enables the removal of calcified plaque material and may promote arterial wall remodeling. This study assessed the technical success, safety, and feasibility of the BYCROSS^®^ atherectomy device in femoropopliteal lesions. **Methods:** This single-center, retrospective cohort study analyzed elective patients undergoing BYCROSS^®^ atherectomy for chronic peripheral arterial disease from March 2022 to May 2023. Patient data, procedural details, and outcomes were retrospectively collected from electronic patient records. The primary performance endpoints of this study were technical success, complications, and patency rates. Primary safety endpoints included 30-day and short-term major adverse limb events (MALEs), major adverse cardiovascular events (MACEs), and mortality rate. **Results:** The study included 19 patients (median age, 71 years; 63% male) with Fontaine class IIb (26%), III (21%), or IV (53%). The BYCROSS^®^ atherectomy device was used to treat 22 limbs in the femoropopliteal tract, of which 11 lesions (50%) were occlusions and 11 were stenoses, with a median length of 24 cm (interquartile range: 17–38). Technical success was achieved in all cases: 4.5% required atherectomy only, 50% required additional balloon angioplasties, 41% required balloon angioplasties and stenting, and 4.5% required segments only stenting. Additional treatment of below-the-knee arteries was performed in 12 patients. Procedurally related complications (not limited to the use of the BYCROSS^®^ device) occurred in 23% of limbs, including distal embolization and laceration. At 30 days, mortality was 5%, the MACE rate was 11%, and the MALE rate was 0%. The observed mortality rate was not directly related to the procedure. Patency (<50% restenosis at duplex ultrasound) was 83% at 30 days. **Conclusions:** The use of the BYCROSS^®^ atherectomy device for the treatment of femoropopliteal lesions appears to be safe and feasible, with high technical success and low MALE and MACE rates in a challenging population with long-segment femoropopliteal lesions. Long-term follow-up in larger patient series is needed to confirm these findings and to determine the durability of this technique.

## 1. Introduction

For patients with chronic limb-threatening ischemia, regaining uninterrupted flow to the below-the-knee and pedal arteries is essential to achieve adequate tissue perfusion and limb salvage [1]. Endovascular techniques have evolved rapidly in recent decades, and due to their minimally invasive character, they have become the preferred treatment over bypass surgery in many centers. Doubts about the optimal treatment strategy remain, however, and several landmark trials comparing an endovascular-first with an open-surgery-first revascularization strategy have led to contradictory results [2,3,4]. A recent systematic review showed that there is no difference in survival, amputation-free survival, reintervention, major amputation, or therapeutic crossover rates between endovascular-first and surgery-first revascularization strategies for CLTI [5].

Endovascular treatments often show limited efficacy in arterial segments with a high atherosclerotic load [6,7,8]. The use of atherectomy devices offers a promising solution because they enable the debulking of plaques, restoration of luminal area, and promotion of arterial wall remodeling [9,10]. Furthermore, preparing the femoropopliteal vessel with atherectomy could potentially improve drug absorption when drug-coated balloons are used and may also reduce the necessity for high-pressure balloon inflation, thereby reducing barotrauma and the subsequent inflammatory response. However, this hypothesis necessitates validation through high-quality research studies [11]. The literature on the routine use of atherectomy devices in femoropopliteal arterial disease is scarce [12], possibly due to the 2011 National Institute for Health and Care Excellence (NICE) guidelines [13], which advise against the use of atherectomy except in clinical trials. In recent years, atherectomy has been increasingly used as a vessel preparation technique. After debulking of the femoropopliteal artery with atherectomy, the working mechanism of drug-eluting stents or balloons is thought to be more effective.

The method of plaque debulking varies among commercially available atherectomy devices and is achieved through mechanisms such as direct cutting, shaving, drilling, or vaporization. Most atherectomy devices come with specific limitations, including the need for wire passage before atherectomy, which carries the risks of subintimal wire positioning, perforation, and distal embolization [12,14]. Additionally, high-speed rotations of the devices may cause mechanical stress on the vessel wall, increasing the risk of complications [6,14,15,16].

The BYCROSS^®^ atherectomy device (Taryag Medical Ltd., Or Akiva, Israel) is a novel rotational atherectomy device that claims to have the advantages of not requiring wire passage of the lesion, using low rotational speed (2000–4500 rpm), having a variable tip size up to 4.7 mm, and pump-mediated aspiration. These properties are thought to increase overall safety and effectiveness in embolic complications, technical success, and initial lumen gain [17]. A prospective multicenter, premarket approval study demonstrated the safety and technical success of the BYCROSS^®^ atherectomy system for patients with infrainguinal arterial stenoses down to the tibioperoneal tract [17]. However, the currently available literature on the performance of the BYCROSS^®^ atherectomy device is scarce.

The present study evaluated the technical success, safety, and feasibility of the BYCROSS^®^ atherectomy device in a single-center cohort of patients with calcified femoropopliteal lesions.

## 2. Materials and Methods

### 2.1. Study Design

This was a single-center, retrospective cohort study of elective patients who underwent treatment with the BYCROSS^®^ atherectomy device in the University Medical Center Groningen (UMCG) from the introduction of the device in March 2022 until May 2023. Clinical follow-up data were included until January 2024, ensuring that a minimum of 6 months of follow-up was available for all patients. The UMCG Institutional Review Board reviewed the study protocol and waived the requirement to obtain informed consent according to the Dutch Medical Research Involving Human Subjects Act. All study procedures adhered to European privacy guidelines and the principles of the Declaration of Helsinki.

### 2.2. Patient Selection

This study included consecutive patients with chronic peripheral arterial disease (PAD) who were treated with the BYCROSS^®^ atherectomy device. The decision for endovascular treatment was made by a multidisciplinary team of vascular surgeons and interventional radiologists according to the standard of care based on the current global vascular guidelines [1]. The criteria for atherectomy treatment were a complex atherosclerotic lesion with mixed plaque or a high calcium load in the native vessels and/or an in-stent stenosis or occlusion. Data on patient demographics, medical history, comorbidities, imaging studies, intraprocedural data, periprocedural complications, and outcomes were obtained from the electronic medical records (G.L.v.L).

### 2.3. Endovascular Revascularization

Endovascular revascularization was performed under local anesthesia in an angiography suite. All procedures were performed in the presence of the same interventional radiologist (J.O.) as the treating physician or as a supervisor. All patients received heparin during the intervention, with a median dosage of 7500 international units (interquartile range: 5000–10,000).

The BYCROSS^®^ atherectomy device was applied according to the instructions for use unless otherwise noted [18]. The device consists of a coaxial, flexible, rotating shaft with an extendable tip, incorporating an aspiration system for removing plaque debris and dislodged thrombotic material. The tip can be expanded, increasing its diameter from 1.9 mm to 4.7 mm. As the shaft rotates, the tip breaks down calcified atheroma or thrombus into small particles, which are simultaneously aspirated into the guide sleeve and collected in an attached bag. After lesion passage and adequate debulking, definitive treatment was performed and could consist of plain old balloon angioplasty (POBA), drug-eluting balloon (DEB) angioplasty, or stenting.

Disease staging was performed using the Global Limb Anatomic Staging System (GLASS) [1]. The Trans-Atlantic Inter-Society Consensus for the Management of Peripheral Arterial Disease (TASC II) was used for the anatomic classification [19].

### 2.4. Outcome Measures

The primary performance endpoints of this study were technical success, 30-day complication rate, and patency. The outcome measures are reported in accordance with the reporting standards of the Society for Vascular Surgery for endovascular treatment of chronic lower-extremity PAD [20] and the 2017 European Society of Cardiology Guidelines on the Diagnosis and Treatment of Peripheral Arterial Diseases [21]. Technical success was defined as a composite of primary and assisted technical successes. Primary technical success was considered achieved if the BYCROSS^®^ atherectomy device, with or without POBA, successfully re-established vessel patency, achieving < 30% residual stenosis on digital subtraction angiography. Assisted technical success was achieved if stenting, resulting in <30% residual stenosis, was necessary to achieve vessel patency. Periprocedural complications were deemed present in the case of distal embolization, arterial laceration or perforation, or bleeding. Technical success and complications were evaluated based on angiographic data by an experienced interventional radiologist (J.O.) with extensive expertise in peripheral endovascular procedures (>100 cases per year). Patency was defined as the absence of restenosis of ≥50% as objectified by duplex ultrasound (DUS).

Primary safety endpoints were an absence of a major adverse limb event (MALE), a major adverse cardiovascular event (MACE), and a 30-day mortality rate. MALE was defined as above-ankle amputation of the index limb or major reintervention. Major reinterventions involved the need for a surgical bypass graft, thrombectomy or thrombolysis, or major surgical graft revisions (e.g., jump graft, interposition graft). Minor reinterventions, not included in the definition of MALE, were collected, and included endovascular procedures (repeat percutaneous transluminal angioplasty, with or without additional stenting) without thrombectomy/thrombolysis [20,22]. MACE was defined as myocardial infarction, stroke, or death from any cause [22].

### 2.5. Statistical Analysis

Study data were collected and managed using Research Electronic Data Capture (REDCap) tools hosted at the UMCG [23,24]. Statistical analysis was performed using SPSS 28 software (IBM Corporation, Armonk, NY, USA). Continuous variables are presented as median and interquartile range (IQR) as appropriate and unless stated otherwise.

## 3. Results

### 3.1. Patients

Between March 2022 and May 2023, 19 patients (22 limbs) with chronic PAD were treated with the BYCROSS^®^ atherectomy device and included in this study. The characteristics of these patients are detailed in Table 1. Three patients received bilateral treatments within the same session. The duration of the procedures was a median of 82 min (IQR: 62–101). Atherectomy of the femoropopliteal lesion was the index intervention; however, in 12 limbs (55%), additional treatment of below-the-knee arteries was performed. Of the atherectomy procedures, 10 (45%) were indicated for reobstructions (1 after POBA and 9 because of in-stent obstructions).

### 3.2. Treatment of Femoropopliteal Lesions

A total of three superficial femoral artery (SFA) lesions, seventeen continuous SFA-popliteal artery (PA) lesions, and two PA lesions were treated. The median lesion lengths were 50 mm (IQR: 30–250) in the SFA, 290 mm (IQR: 173–380) in the SFA-PA, and 81 mm (range: 51–110) in the PA lesions. Occlusions were present in one SFA lesion (33%), eight SFA-PA lesions (47%), and two PA lesions (100%), with the remaining lesions classified as stenoses. Detailed characteristics of these lesions, including TASC classification, are summarized in Table 2.

The BYCROSS^®^ atherectomy device was used in all 22 femoropopliteal lesions. Technical success was 100%. Atherectomy was the sole treatment for only one lesion (4.5%), whereas most of the lesions underwent additional treatment with POBA (50%), POBA and stenting (41%), or stenting (4.5%), as detailed in Table 2. Bailout stenting was deemed necessary for 10 lesions (45.5%). The reasons for stenting were flow-limiting dissection (*n* = 5), recoil (*n* = 3), extravasation (*n* = 1), and in-stent stenosis (*n* = 1). Complications occurred in five limbs (23%): three involved (micro)embolization requiring thrombolysis, thrombectomy, or thrombosuction, and two involved lacerations, with one requiring stenting and the other prolonged balloon angioplasty. The BYCROSS^®^ device was used according to the instructions for use in 19 limbs (86%). However, off-label treatment was performed in three limbs: first, for creating a subintimal canal; second, for facilitating retrograde passage through the lesion; and third, for treating a calcified stenosis of the common femoral artery (CFA). A complication occurred during the off-label treatment of the CFA, where the sheath extended, causing a CFA laceration. The laceration was addressed with prolonged balloon dilatation, resulting in a small false aneurysm. After this intervention, the vessel was patent, allowing for further antegrade treatment. The small false aneurysm dissolved during follow-up.

### 3.3. Treatment of Adjunctive Lesions

In 12 limbs, additional infrapopliteal lesions were treated (Table 3). Five lesions were treated using the BYCROSS^®^ atherectomy device (one proximal anterior tibial artery lesion, one posterior tibial artery [PTA] lesion, and three tibiofibular trunk lesions) and had a technical success rate of 100%. During one procedure in which the BYCROSS^®^ atherectomy device was used for infrapopliteal lesions, distal embolization occurred, which required thrombosuction, but assisted technical success was still obtained.

### 3.4. Thirty-Day Outcomes

At 30 days, the MACE rate was 11% (*n* = 2), the MALE rate was 0%, and 30-day mortality was 5% (*n* = 1). The patient who died did so 13 days after the intervention as a result of multiple organ failure, including renal failure, with a palliative trajectory. One patient had an ischemic cerebrovascular accident of the right hemisphere 2 days after the intervention.

Eighteen limbs (82%) had a 30-day follow-up with DUS, with a median follow-up of 42 days (IQR: 9–48). The 30-day patency (<50% restenosis at duplex ultrasound) of these limbs was 83%.

### 3.5. Short-Term Outcomes

Short-term follow-up was 353 days (IQR: 253–605). The mortality rate was 16% (*n* = 3), the MACE rate was 21% (*n* = 4), and the MALE rate was 14% (*n* = 3). One patient died 75 days after the intervention of cardiogenic shock with a palliative trajectory. Another patient died after 256 days of end-stage renal failure, for which dialysis was intentionally stopped.

The MALEs consisted of two below-the-knee amputations performed after reocclusion of the femoropopliteal segment. Another patient required femoropopliteal bypass surgery and amputation of toes due to reocclusion.

Minor endovascular reinterventions (PTA and stenting) were performed in three limbs to obtain patency of the treated lesion. Additional digital amputations were performed in two limbs due to infection and gangrene with patent vasculature.

Nine limbs (47%) underwent further follow-up with DUS, with a median of 307 days (IQR: 276–340). The short-term patency of these limbs was 78%.

## 4. Discussion

This single-center retrospective study performed in a real-world setting presents the technical success and short-term outcomes of the BYCROSS^®^ atherectomy device used for long-segment femoropopliteal lesions. The safety and feasibility of the device were demonstrated, with a technical success rate of 100%, a 30-day mortality of 5%, a 30-day MACE rate of 11%, and a 30-day MALE rate of 0%, in a cohort of patients with significant comorbidities (i.e., 75% with American Society of Anesthesiologists Physical Status Classification (ASA) III and IV). The patency rate at 30 days was acceptable at 83%.

These findings correspond well with the prospective multicenter, premarket approval study by Tessarek et al. [17]. In a reasonably comparable cohort of patients, the authors report a procedural success rate of 95%, where procedural success was defined as residual stenosis of ≤50% after atherectomy alone and as ≤30% on completion of angiography achieved by atherectomy, angioplasty, and/or stenting and the absence of serious adverse events. Major adverse events occurred in 4 of 41 patients (10%) within 30 days and one additional event at 6 months of follow-up. A post-atherectomy PTA was performed in 39 of 41 lesions (95%), and stenting was performed in 12 of 41 lesions (29%) after atherectomy. In their study, the authors reported passage of the BYCROSS^®^ atherectomy device without wire passage of the lesions in 11 of 41 lesions (27%), compared with 3 of 22 lesions (14%) in our present study. It is important to note that the premarket study cohort might represent a more selected group of patients with fewer comorbidities due to the more stringent inclusion and exclusion criteria applied for their study [17].

During follow-up, three patients died, due to cardiac failure, multiorgan failure (including renal failure), and renal failure, respectively. Research conducted on patients undergoing valve replacement suggested that elevated preoperative NT-proBNP levels may correlate with an increased risk of postoperative acute kidney injury [25]. Another study indicated that cardiac biomarkers, including NT-proBNP, offer prognostic insights, including mortality prediction, in patients with PAD [26]. In the current study, NT-proBNP values were determined in only three patients, so no conclusions could be made. It seems worthwhile to include an assessment of NT-proBNP in future intervention studies in PAD patients.

In the current study, periprocedural complications occurred in 23% of the limbs. Distal embolization occurred in three limbs (14%) and is higher than reported in the premarket study by Tessarek et al. [17]. In the context of the complications observed in this study, discerning the specific cause of the complication is challenging. It could be associated with BYCROSS^®^ atherectomy and also with the additional PTA or stent placement, especially in patients with additional below-the-knee interventions. This makes it difficult to attribute the adverse outcome to a single procedural element. Prior research showed no difference in rates of embolization between atherectomy and balloon angioplasty groups [10], but atherectomy devices have been associated with distal embolization [27,28]. Unlike some other atherectomy devices, the design and functionality of the BYCROSS^®^ atherectomy device were considered to obviate the necessity for distal embolic protection in this study because its low velocity and high aspiration capacity might be effective in preventing thrombotic events [17,18]. Several protection devices are available, and in other atherectomy devices, their use has been recommended [12,29]. Furthermore, the use of an embolic protection device is advised for chronic total occlusions, in-stent stenoses, thrombotic lesions, calcified lesions > 40 mm, and atherosclerotic lesions > 140 mm [30]. Since in this patient series a relatively high distal embolization rate was found, and high-risk lesions were treated, the use of distal embolic protection should be considered in future studies with the BYCROSS^®^ atherectomy device.

Two meta-analyses of conventional PTA and atherectomy compared with PTA in patients with femoropopliteal lesions were recently published [9,10]. The first study, including four randomized controlled trials (RCTs), found increased technical success for atherectomy and PTA compared with PTA (risk ratio [RR], 0.22; 95% confidence interval [CI], 0.13–0.38; *p* < 0.001; I^2^ = 0%; high quality), and reduced rates of bailout stenting (RR, 0.15; 95% CI, 0.07–0.32; *p* < 0.001; I^2^ = 16%; high quality) and flow-limiting dissection (RR, 0.24; 95% CI, 0.13–0.47; *p* < 0.001; I^2^ = 0%; high quality), with no significant differences in primary patency, target lesion revascularization, mortality, and major adverse event rates at the 1-year follow-up [9]. Technical success in the trials ranged from 87% to 95% for the atherectomy groups, whereas bailout stenting was performed in 0% to 7% of patients in the included RCTs. The pooled primary patency rate at 1 year was 81% in the atherectomy group.

The second meta-analysis, including six RCTs, found increased primary patency at 1 year (odds ratio, 2.04; 95% CI, 1.14–3.62), reduced major amputation rates (mean difference, 2.01; 95% CI, 0.05–0.77, *p* = 0.02), and decreased rates of bailout stenting (odds ratio, 0.07; 95% CI, 0.02–0.25; *p* = 0.001). In this study, no significant differences in target lesion revascularization, MACE, and distal embolization were observed [10]. The primary patency rate at 1 year in three of the trials ranged from 67% to 80% for the atherectomy groups. Bailout stenting was performed in 0% to 6% of patients in the atherectomy group. The major amputation rate at 1 year in the atherectomy group varied between 0% and 8%. Besides the higher rate of bailout stenting in the present study, these results correspond well. The higher rate of bailout stenting in the present study compared with prior studies [9,10,17] might be due to the retrospective nature of the study, in which no strict inclusion criteria could be maintained, or the learning curve of the treating intervention radiologist, since these are the first results of the use of the device in our center.

The BYCROSS^®^ device is based on rotational atherectomy. Other types of atherectomy are designed to cut, shave, drill, or vaporize [12,14,31]. The preferred type of atherectomy device may be dependent on lesion characteristics. Rotational atherectomy, for instance, is mostly used in calcified lesions in both peripheral and coronary arteries [31]. Studies comparing different atherectomy devices are scarce and exhibit heterogeneity regarding lesion characteristics, indication for use, methodology, and reporting outcomes, with no clear preferences for a particular device [32,33,34]. It would be beneficial to compare the various types of atherectomy devices, including technical and clinical outcomes, in a more homogenous and well-powered study.

The present study has some limitations, and the retrospective design warrants careful interpretation of our findings. First, the retrospective nature of the study makes selection bias more likely. Inclusion was arbitrary, including all patients with chronic PAD treated with the BYCROSS^®^ atherectomy device. Another study limitation is the lack of a proper control group and the small sample size with short-term follow-up. The available follow-up is too short to determine the efficacy of the treatment and the durability of the results that were reported. Finally, there was considerable clinical heterogeneity in lesion characteristics, clinical disease stage (i.e., both intermittent claudication and chronic limb-threatening ischemia), and adjunctive treatments of other segments in this cohort of patients.

Further high-quality studies comparing different endovascular strategies with and without atherectomy devices or comparing different endarterectomy devices for each vascular segment alongside cost-effectiveness analyses are needed to determine the definitive place of this atherectomy device in the endovascular armamentarium.

## 5. Conclusions

The use of the BYCROSS^®^ atherectomy device for the treatment of long-segment femoropopliteal lesions appears to be safe and feasible, with high technical success and low 30-day MALE and MACE rates in a small single-center retrospective patient series. Long-term follow-up in a larger series is needed to confirm these findings and to determine the durability of the results.

## Figures and Tables

**Table 1:** Patient characteristics.

Variable	N Patients (Total *n* = 19)
Age (years)	71 (65–78)
Male sex	12 (63%)
Body mass index	26.2 (22.2–30.2)
Renal insufficiency *	8 (42%)
Diabetes	11 (58%)
Hypercholesterolemia	9 (47%)
Heart failure	12 (63%)
Hypertension	13 (68%)
COPD	4 (21%)
CVA/TIA	5 (26%)
Current smoker	6 (32%)
ASA	
II	4 (21%)
III	10 (53%)
IV	4 (21%)
*Missing*	1 (5%)
Fontaine class **	
IIb	5 (26%)
III	4 (21%)
IV	10 (53%)
Anticoagulant use	19 (100%)
Antiplatelet agents	12 (63%)
Direct oral anticoagulant	6 (32%)
Vitamin K antagonist	1 (5%)
GLASS stage ***	
I	2 (10.5%)
II	2 (10.5%)
III	15 (79%)

Data are presented as frequency (%) or as median (interquartile range). * Estimated glomerular filtration rate < 60 mL/min/1.72 m^2^. ** Patients treated for 2 limbs had similar Fontaine stages in both limbs. *** Patients who underwent treatment for 2 limbs had similar GLASS stages in both limbs. COPD = chronic obstructive pulmonary disease; CVA = cerebrovascular accident; TIA = transient ischemic attack; ASA = American Society of Anesthesiologists; GLASS = Global Limb Anatomic Staging System.

**Table 2:** Femoropopliteal lesion characteristics.

	SFA	SFA-PA	PA	Total
Number	3	17	2	22
Length (mm)	50 (range: 30–250)	290 (IQR: 173–380)	81 (range: 51–110)	238 (IQR: 110–353)
TASC score				
• TASC A	2 (67%)	1 (6%)	-	3 (14%)
• TASC B	-	3 (18%)	1 (50%)	4 (18%)
• TASC C	1 (33%)	9 (53%)	-	10 (45%)
• TASC D	-	4 (24%)	1 (50%)	5 (23%)
Occlusion	1 (33%)	8 (47%)	2 (100%)	11 (50%)
Type of treatment				
• BYCROSS^®^ only		1 (6%)		1 (4.5%)
• BYCROSS^®^ + PTA	2 (67%)	8 (47%)	1 (50%)	11 (50%)
• BYCROSS^®^ + stent		1 (6%)		1 (4.5%)
• BYCROSS^®^ + PTA + stent	1 (33%)	7 (41%)	1 (50%)	9 (41%)
Guidewire used	3 (100%)	15 (88%)	1 (50%)	19 (86%)
Technical success	3 (100%)	17 (100%)	2 (100%)	22 (100%)
• Primary technical success	2 (100%)	9 (100%)	1 (100%)	12 (55%)
• Assisted primary technical success	1 (100%)	8 (100%)	1 (100%)	10 (45%)
Complications	-	5 (29%)	-	5 (23%)

Data presented as median (IQR or range, depending on the number of observations) or frequency (%). SFA = superficial femoral artery; SFA-PA = continuous lesion of superficial femoral artery and popliteal artery; PA = popliteal artery; TASC = Trans-Atlantic Inter-Society Consensus for the Management of Peripheral Arterial Disease; PTA = percutaneous transluminal angioplasty.

**Table 3:** Infrapopliteal lesions.

	ATA	PTA	Peroneal	DPA	TFT
Number	12	6	3	2	9
Length (mm)	160.58 *± 121.1*	261.7 *± 146.5*	50.0 *± 45.8*	47.5 *± 31.8*	21.7 *± 13.2*
TASC score					
• TASC A	3 (25%)	1 (17%)	2 (67%)	1 (50%)	9 (100%)
• TASC B	1 (8%)	-	-	1 (50%)	-
• TASC C	4 (33%)	4 (67%)	1 (33%)	-	-
• TASC D	4 (33%)	1 (17%)	-	-	-
Type of treatment					
• BYCROSS^®^ only	-	-	-	-	-
• BYCROSS^®^ + stent	-	-	-	-	-
• BYCROSS^®^ + PTA	1 (8%)	1 (17%)	-	-	3 (33%)
• BYCROSS^®^ + PTA + stent	-	-	-	-	-
• PTA only	9 (75%)	5 (83%)	2 (67%)	2 (100%)	6 (67%)
• PTA + stent	-	-	-	-	-
• Untreated	2 (17%)	-	1 (33%)	-	-
Technical success	10 (83%)	6 (100%)	2 (67%)	2 (100%)	9 (100%)

Data are presented as mean ± standard deviation or frequency (%). ATA = anterior tibial artery; PTA = posterior tibial artery; DPA = dorsalis pedis artery; TFT = tibial fibular trunk; TASC = Trans-Atlantic Inter-Society Consensus for the Management of Peripheral Arterial Disease; PTA = percutaneous transluminal angioplasty.

## Data Availability

The data presented in this study are available on request from the corresponding author.
